# Enterovirus D68 molecular and cellular biology and pathogenesis

**DOI:** 10.1016/j.jbc.2021.100317

**Published:** 2021-01-21

**Authors:** Matthew J. Elrick, Andrew Pekosz, Priya Duggal

**Affiliations:** 1 Department of Neurology, Johns Hopkins School of Medicine, Baltimore, Maryland, USA; 2 Department of Molecular Microbiology and Immunology, Johns Hopkins Bloomberg School of Public Health, Baltimore, Maryland, USA; 3 Department of Epidemiology, Johns Hopkins Bloomberg School of Public Health, Baltimore, Maryland, USA

**Keywords:** enterovirus, acute flaccid myelitis, virus replication, innate immunity, adaptive immunity, AFM, acute flaccid myelitis, CDC, US Centers for Disease Control and Prevention, COVID-19, coronavirus disease 2019, eIF4, eukaryotic initiation factor 4, EMG, electromyography study, EV-A71, enterovirus A71, EV-D68, enterovirus D68, GI, gastrointestinal, hnRNP, heterogenous nuclear ribonucleoprotein, ICAM, intercellular adhesion molecule, iPS, induced pluripotent stem cell, IRES, internal ribosome entry site, IVIG, intravenous immunoglobulin, MDA, melanoma differentiation–associated protein, NCS, nerve conduction study, RIG, retinoic acid–inducible gene, TLR, Toll-like receptor

## Abstract

In recent years, enterovirus D68 (EV-D68) has advanced from a rarely detected respiratory virus to a widespread pathogen responsible for increasing rates of severe respiratory illness and acute flaccid myelitis (AFM) in children worldwide. In this review, we discuss the accumulating data on the molecular features of EV-D68 and place these into the context of enterovirus biology in general. We highlight similarities and differences with other enteroviruses and genetic divergence from own historical prototype strains of EV-D68. These include changes in capsid antigens, host cell receptor usage, and viral RNA metabolism collectively leading to increased virulence. Furthermore, we discuss the impact of EV-D68 infection on the biology of its host cells, and how these changes are hypothesized to contribute to motor neuron toxicity in AFM. We highlight areas in need of further research, including the identification of its primary receptor and an understanding of the pathogenic cascade leading to motor neuron injury in AFM. Finally, we discuss the epidemiology of the EV-D68 and potential therapeutic approaches.

Enterovirus D68 (EV-D68) belongs to the Picornaviridae family and the genus *Enterovirus*. Picornaviruses are characterized by a single-stranded positive-sense RNA ((+)-ssRNA) genome that is carried by an icosahedral capsid without an envelope. EV-D68 has gathered interest recently because of its increasing prevalence and its association with infections causing high morbidity predominantly in children. These complications include severe respiratory infection and the poliomyelitis-like disorder acute flaccid myelitis (AFM). Other enteroviruses, including enterovirus A71 (EV-A71) and coxsackievirus A16, have also been associated with AFM at lower frequencies (1, 2, 3).

Little was known about EV-D68 prior to its first major outbreak in 2014. However, picornaviruses in general have been well studied since the identification of poliovirus as the cause of recurrent epidemics of poliomyelitis (4). Much of what is known about EV-D68 therefore derives from common principles determined from other human pathogens in the picornavirus family. Recent work has evaluated specific aspects of EV-D68 biology. The field has begun to draw comparisons and contrasts between EV-D68 and other picornaviruses and investigated how these characteristics may underlie its unique biology.

In this review, we will discuss the life cycle and molecular biology of enteroviruses in general as a framework upon which to understand EV-D68. Within that context, we will highlight what is known about EV-D68. We will identify areas in which contemporary strains of EV-D68 differ from its historic prototypes and from other enteroviruses and discuss how these features may lead to neurologic complications in AFM. We will discuss the molecular evolution underlying these features and how they contribute to the virology of EV-D68 and its influences on the biology of host cells. Finally, we will place these findings into the context of the epidemiology of and potential therapeutic approaches for AFM and identify areas in need of further research.

## History and taxonomy

The genus *Enterovirus* comprises the four human enterovirus species A to D, three human rhinovirus species A to C, as well as enterovirus species E to L that infect nonhuman hosts. Enteroviruses were initially classified based on their clinical syndrome and subtyped based on serology, leading to the traditional names of *Enterovirus* genus members such as poliovirus and coxsackievirus. These multiple species were later reclassified based on genomic taxonomy. In the current classification scheme, the three poliovirus subtypes belong to the *Enterovirus C* species, coxsackieviruses, are distributed across species A to C, and the remainder retain the name enterovirus. Since 1970 and beginning with enterovirus 68, newly discovered enterovirus subtypes have been numbered sequentially by their order of discovery. Following the reclassification, these numbers were appended to the species letter name. For example, enterovirus 68 became EV-D68 (5).

**Figure 1:**
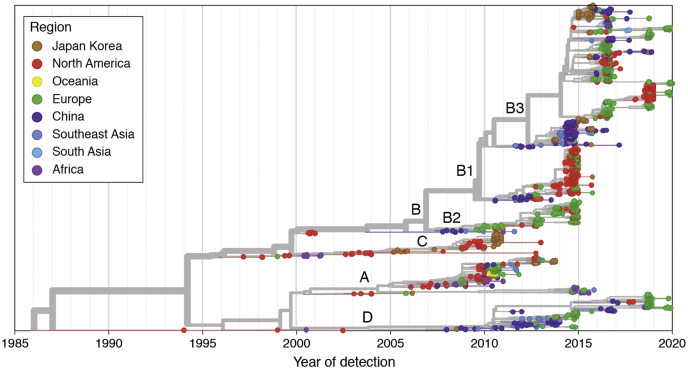
**Phylogenetic tree of enterovirus D68 clades.** Year of detection is represented on the *x*-axis. The region in which each isolate was detected is signified by color as per the key in the *upper left*. Clade names are labeled at the branch points. Visualization of phylogenetic data was performed using Nextstrain (230).

EV-D68 was first isolated in 1962 from pharyngeal swabs of four children hospitalized for severe acute lower respiratory tract illness, designated the Fermon, Rhyne, Franklin, and Robinson strains. Fermon continues to be considered the prototypic strain of EV-D68 (6). A separate strain was classified as rhinovirus 87 in 1963 because of strong phenotypic similarity to rhinoviruses, until genomic data revealed that it was also an enterovirus 68 strain, and these were most appropriately classified under the *Enterovirus D* species (7, 8). There are four identified EV-D68 clades (A, B, C, and D), which have been separated primarily based on sequencing of the VP1 gene encoding a key component of the viral capsid. Clades A and B are prevalent globally across the United States, Europe, and Asia. Clade D separated from clade A, which shares a common ancestor. Clades C and D are also present throughout these regions but at lower frequencies (9, 10, 11, 12, 13) (Fig. 1).

## Molecular virology

### Genome organization

All picornaviruses, including EV-D68, share a common genome organization. The (+)-ssRNA genome consists of a 5′-UTR containing an internal ribosome entry site (IRES) and a spacer region, an open reading frame encoding a single precursor polypeptide, and a 3′-UTR with a poly-A tail. The polypeptide undergoes post-translational proteolytic processing to yield structural proteins, VP1, VP2, VP3, and VP4, and nonstructural proteins, 2A, 2B, 2C, 3A, 3B, 3C, and 3D (14).

### Capsid structure

**Figure 2:**
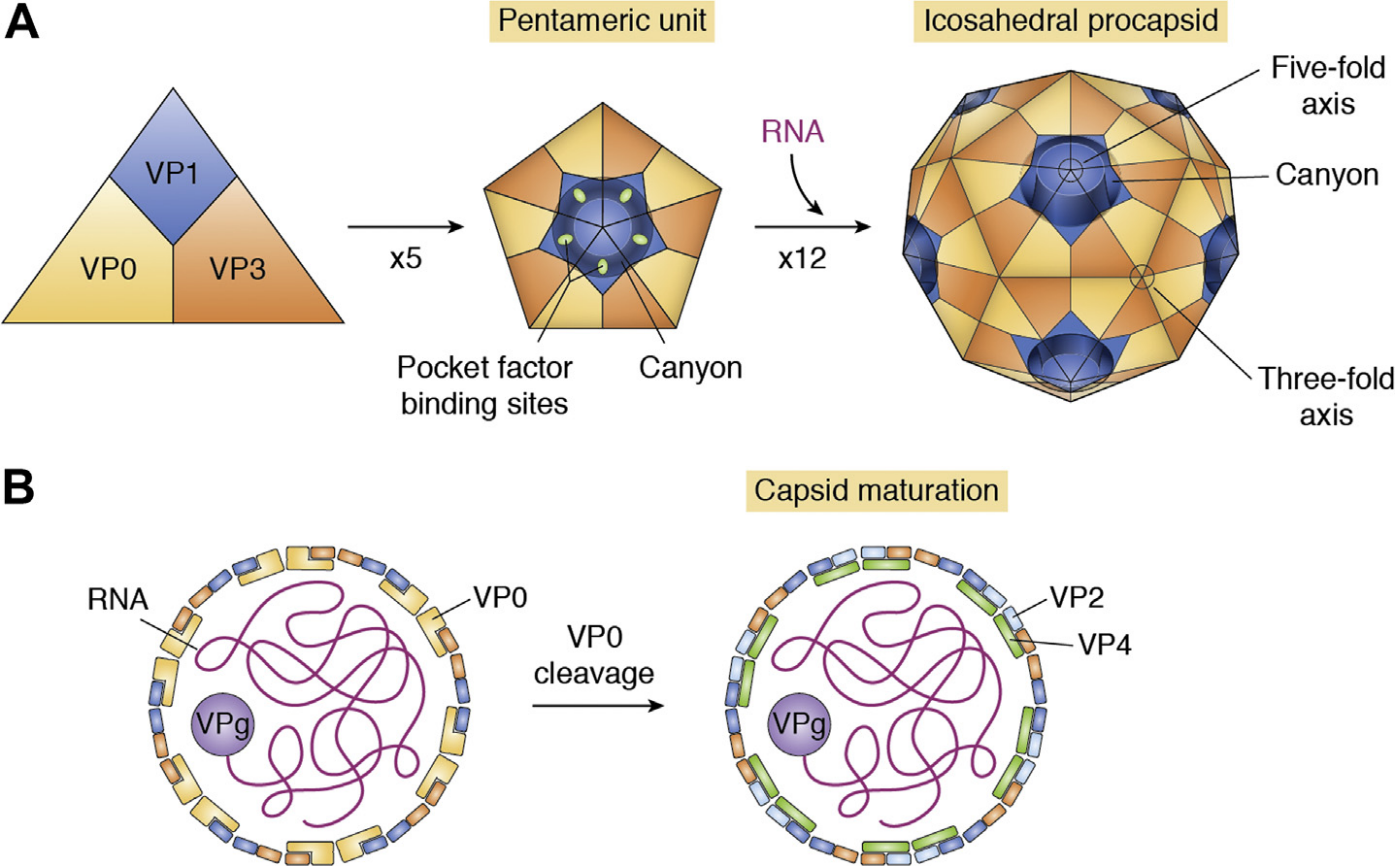
Assembly of the enterovirus D68 capsid. *A*, Structural proteins VP0, VP1, and VP3 self-assemble into a trimeric structure that forms the basic building blocks of the capsid. Five of these units next assemble into a pentamer. Twelve pentameric units assemble into the procapsid, an icosahedral structure with 60 sides. This structure contains alternating vertices of fivefold and threefold symmetry. Surrounding the fivefold vertex lies a canyon. Within each VP1 subunit in the canyon is a host-derived small hydrophobic molecule, known as the pocket factor. The exact identity of the pocket factor in enetrovirus D68 is not known. *B*, cross section of the virion showing VPg-bound (+)-ssRNA in the interior, which is loaded into the capsid during the assembly process. The orientation of VP peptides with respect to the interior and exterior faces of the capsid is schematically represented. During maturation of the capsid, VP0 is cleaved to generate VP2 and VP4. In the mature virion, VP4 is oriented primarily toward the interior, whereas VP1, VP2, and VP3 are oriented primarily toward the outer face.

The enterovirus capsid, consisting of the four structural proteins VP1 to VP4, forms a nonenveloped icosahedral structure consisting of 60 copies of each protein. They are arranged in subunits with VP1 to VP3 facing outward and VP4 lining the interior of the capsid, with the vertices coming together to form unions of alternating threefold and fivefold symmetry (15, 16). Surrounding the fivefold vertex lies a “canyon” thought to play an important role in receptor binding (15, 17). At the base of the canyon, there is a hydrophobic pocket within each VP1 subunit containing a host-derived lipid-like “pocket factor.” Crystallography studies have confirmed that these structural features are also present in EV-D68 (18, 19) (Fig. 2)

### Viral receptors and host cell binding

To initiate infection, the viral capsid must recognize and enter a host cell. The first step in this process is attachment, wherein the viral capsid binds to attachment factors on the host cell, which often consist of carbohydrate moieties. This attachment step serves to concentrate virions at the cell surface. The next step is binding of a receptor, most commonly an integral membrane protein, and this event mediates the entry of the virus into the cell *via* receptor-mediated endocytosis (20). The first identified and best characterized of picornavirus receptors is CD155 for poliovirus (21). However, there is a wide range of diversity for receptor usage among picornaviruses (reviewed in Wen *et al.* [22]).

The first hypothesized receptor for EV-D68 was sialic acid (or a sialylated glycoprotein). This is because historical strains of EV-D68 had marked reduction of viral binding to the cell surface following treatment with neuraminidase, which removes sialic acid (23). More recent studies have identified binding of α2,6- and α2,3-linked sialic acids by Fermon and several EV-D68 strains from the years 2010 to 2011 and have shown that sialic acid binding displaces the pocket factor (18, 24).

Interestingly, some strains dating from 2012 onward are capable of sialic acid–independent binding. These strains retain their sialic acid–binding capacity, suggesting that an additional attachment factor or receptor binding mechanism is present (25). Three contemporary clade B strains from the 2014 US outbreak were capable of sialic acid–independent infection of human-induced pluripotent stem cell (iPS)–derived motor neurons, whereas Fermon, Rhyne, and a clade A strain were not (26). A separate study showed that Fermon is in fact capable of sialic acid–independent infection in human and mouse cell lines but requires longer incubation times and infects with lower efficiency than when sialic acid is left intact (27). In the absence of sialic acid, EV-D68 may utilize sulfated glycosaminoglycans instead (28).

Intercellular adhesion molecule 5 (ICAM-5) has also been proposed as an EV-D68 receptor. ICAM-5 is a cell adhesion molecule expressed on neurons (29). The related protein ICAM-1 is a well-characterized receptor for multiple rhinoviruses and coxsackieviruses (22). Studies in cultured human cell lines demonstrate enhanced viral replication in the presence of ICAM-5, for both sialic acid–dependent and sialic acid–independent EV-D68 strains (30). However, the physiologic relevance of this finding has been questioned, as the human respiratory tract and spinal cord do not express ICAM-5 *in vivo* (26).

These data show that sialic acid, sulfated glycosaminoglycans, and ICAM-5 may potentiate infection by EV-D68 strains to varying degrees but are not required for infection. Any of these molecules may be attachment factors or coreceptors. However, the primary receptor utilized for productive infection of human patients by EV-D68 remains unknown.

### Viral internalization and uncoating

In general, receptor binding of enteroviruses is followed by structural reorganization of the viral capsid, leading to formation of the “A particle,” an enlarged intermediate in which VP4 and the N-terminal portion of VP1, previously oriented toward the interior of the capsid, are externalized (31, 32). The A particle then enters the host cell *via* endocytosis (33). Inside the endosome, viral capsid proteins reorganize again to form a pore in the endosomal lipid bilayer, through which the viral genome is delivered into the cytoplasm (34, 35). Uncoating of enteroviral particles may require only the presence of its receptor, while some strains also require an acidic environment, as is present following endosome maturation (36). EV-D68 has been shown to be exquisitely acid sensitive, with the transition to A particle and pore-containing particle induced by exposure to pH 6.0 *in vitro* (37). The uncoating of many picornaviruses also requires the host phospholipase PLA2G16, *via* an unknown mechanism (38, 39). However, a sialic acid–independent EV-D68 strain isolated from the 2012 outbreak in the Netherlands did not require PLA2G16 when utilizing sulfated glycosaminoglycan as its receptor and also did not require endosomal acidification for uncoating (28).

### Temperature dependence and acid sensitivity

Enteroviruses have been traditionally identified based on physiologic characteristics and serology. Classical enteroviruses from species A to C replicate best at temperatures near 37 °C and are able to tolerate acidic conditions, consistent with their requirement to traverse the gastrointestinal (GI) tract to achieve infection. By contrast, rhinoviruses typically replicate most efficiently at lower temperatures approximating that of the nasopharynx and are acid labile (40). EV-D68 is unusual in this respect. Although its genetic and antigenic properties are most similar to other enteroviruses, its prototypical strains were found to have an optimum replication temperature of 33 °C and acid lability (6). EV-D68 is therefore more phenotypically similar to rhinoviruses, and accordingly, it is transmitted by respiratory droplets and produces a predominantly respiratory syndrome in most patients. While the prototypical strains of EV-D68 show attenuated growth at higher temperatures, multiple strains isolated in 2014 and later replicate with equal efficiency at 32 °C or 37 °C (41). These changes may contribute to the apparent increased propensity for newer EV-D68 strains to establish systemic infection and neuroinvasion.

### Translation of the viral genome

Translation of the enteroviral RNA genome begins immediately following its delivery to the cytosol. The process circumvents the requirement for a 5′ cap, instead utilizing the IRES located within the 5′-UTR (42). The 3′ poly-A tail is bound by host poly-A binding protein 1, which also interacts with eukaryotic initiation factor 4G (eIF4G) bound to the IRES, thus forming a circular structure that resembles host cell mRNA (43). Translation requires additional host-derived translation-initiating factors, including eIF2, eIF3, eIF4A, eIF4B, eIF1A, and the C-terminal portion of eIF4G. The process also requires host IRES transactivating factors, including polypyrimidine tract–binding protein 1 (also known as heterogenous nuclear ribonucleoprotein [hnRNP I]), poly(rC) binding protein 2 (also known as hnRNP E2), hnRNP A1, lupus antigen La protein, and serine/arginine-rich splicing factor 3. Collectively, these factors recruit the translation initiation complex to the viral IRES (reviewed in Sweeney *et al.* and Flather *et al.* [44, 45]).

The enteroviral genome is translated into a single polypeptide that undergoes proteolytic processing to yield its four structural and seven nonstructural proteins. The first proteolytic event is self-cleavage at the amino terminus of 2A^pro^ by its chymotrypsin-like protease activity, separating the structural proteins VP1 to VP4 from the remainder of the polypeptide. Further proteolytic processing is performed by the 3CD^pro^ and 3C^pro^ proteases, yielding structural peptides VP0, VP1, and VP3, and series of intermediate and mature nonstructural peptides. Finally, VP2 and VP4 are generated from VP0 through a self-cleavage event during maturation of the viral capsid (46).

Enteroviruses undergo a “switch” that ends the translation-predominant phase of their life cycle. This switch occurs because of cleavage of polypyrimidine tract–binding protein 1 (47), poly(rC)-binding protein 2 (48, 49), and poly-A–binding protein 1 (50) by 3C^pro^. The switch occurs only when 3C^pro^ has reached a sufficiently high concentration, and therefore, this event acts as a sensor for the accumulation of viral proteins. Beyond this point, RNA replication predominates.

### Genome replication

RNA replication is undertaken by the enteroviral RNA-dependent RNA polymerase, 3D^pol^. It first generates negative sense (−)-ssRNAs. These form the template for production of multiple copies of genomic (+)-ssRNA. The process is stimulated by circularization of (−)-ssRNA induced by binding of host hnRNP C1/C2 (51). Negative strand synthesis and RNA translation cannot occur concurrently, presumably because ribosomes and RNA polymerase are unable to traverse viral RNA in opposite directions simultaneously (52).

Enterovirus RNA replication takes place on replication organelles, single membrane– and double membrane–bound vesicles thought to be derived from the endoplasmic reticulum–Golgi apparatus and/or autophagosomes with additional fatty acid contributions from lipid droplets (53, 54, 55). Performing RNA translation at the surface of these organelles is thought to promote the colocalization of RNA-binding proteins and may also assist with evasion of host RNases (56, 57, 58).

### Assembly and release of viral particles

The assembly of enteroviruses occurs in the cytoplasm following RNA replication. VP0, VP1, and VP3 form a trimeric structure, which then self-assemble into a pentameric unit. Twelve of these units come together to form the icosahedral enterovirus capsid (15). Newly synthesized (+)-ssRNA is covalently linked to VPg (derived from protein 3B) at the 5′ end and then loaded into the capsid (59). The process of capsid maturation in EV-D68 is promoted by enclosure in autophagosome-derived vesicles (60). As vesicles mature and acidify, the cleavage of VP0 into VP2 and VP4 is stimulated (Fig. 2).

**Figure 3:**
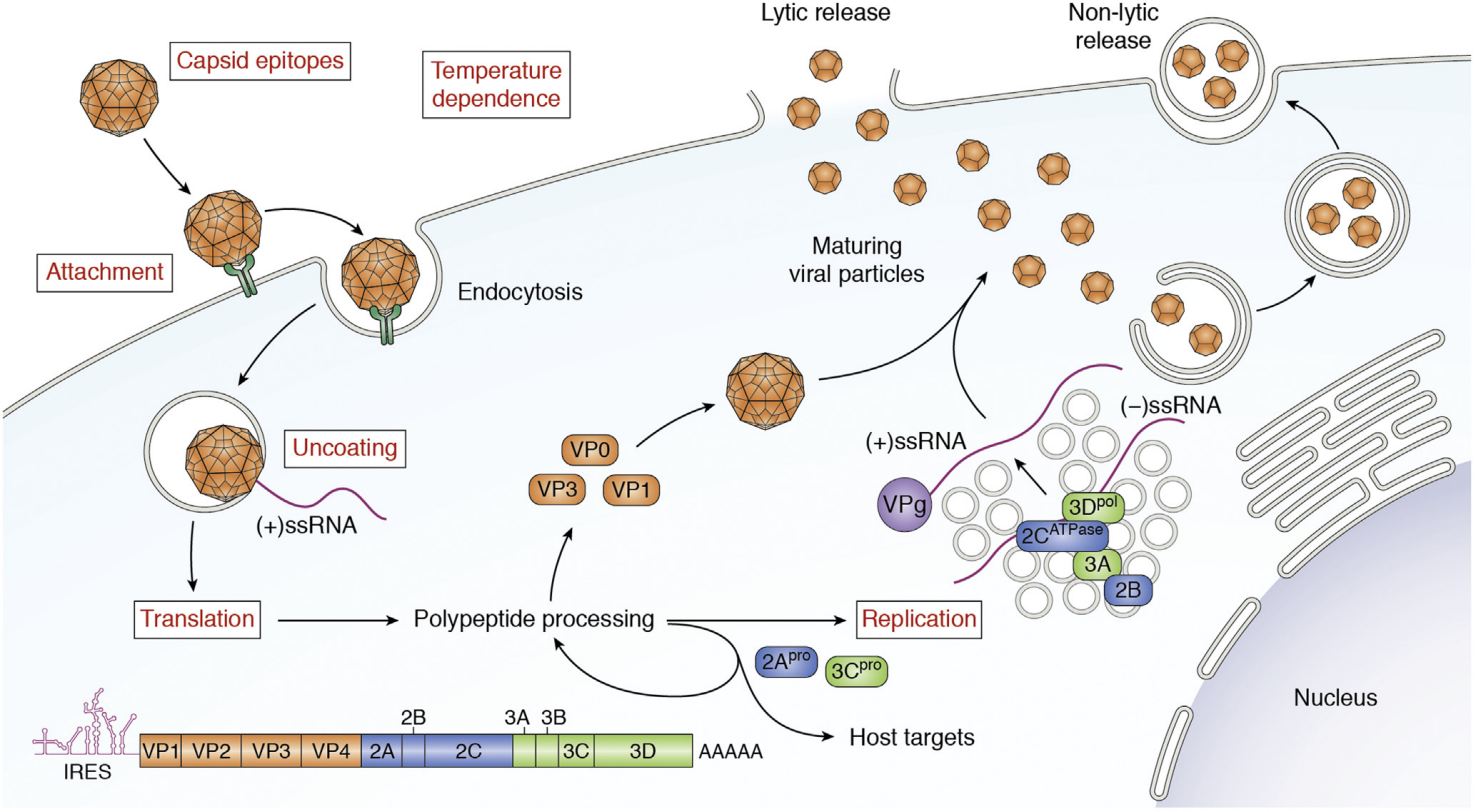
**Life cycle of enterovirus D68 (EV-D68).** The mature EV-D68 virion attaches to the plasma membrane of the host cell and then undergoes receptor-mediated endocytosis. The capsid then undergoes uncoating, in which a rearrangement to create a pore through the endosomal membrane through which viral (+)-ssRNA enters the cytoplasm. From here, the viral genome is translated into a polypeptide that undergoes further proteolytic processing to generate structural and nonstructural proteins. In addition, (−)-ssRNA is generated by RNA replication, which occurs on vesicular structures known as replication organelles. These RNAs become the template for new copies of the (+)-RNA genome. Virions assemble from structural proteins and VPg-linked RNA as detailed for Figure 2. These immature viral particles are largely taken up by autophagosomes, within which the acidic environment stimulates maturation of the capsid. Mature virions are released either by exocytosis of these autophagic vesicles or by cell lysis and release of nonenveloped viral particles. *Red text* indicates steps of the enterovirus life cycle in which contemporary EV-D68 strains have been shown to differ from other enteroviruses and/or from historical strains of EV-D68.

Finally, mature viral particles may exit the cell by either cell lysis or exocytosis of autophagosome-derived vesicles containing multiple viral particles each. During EV-D68 infection, maturation of virus-containing autophagosomes is prevented by 3C^pro^-mediated cleavage of SNARE proteins that would typically promote autophagosome to endosome/lysosome fusion. Instead, these vesicles are redirected to the cell surface and undergo exocytosis (60). A recent study in poliovirus showed that similar exosome-like particles contain multiple mature virions, positive- and negative-sense nonencapsidated viral RNA, nonstructural viral proteins, and several host proteins. These particles are infectious and in fact initiate viral replication more rapidly than equivalent numbers of nonmembrane-enclosed virus (61). This mechanism may allow for increased efficiency of cell-to-cell spread within the infected host as well as immune evasion by shielding the antigenic viral capsid proteins within the vesicle (Fig. 3).

## Molecular evolution of contemporary strains

EV-D68 has undergone significant genetic changes since the original identification of its prototypical strains. As discussed in more detail later, the primary regions of variation include the VP1 capsid protein, the 5′UTR, and several nonstructural proteins. VP1 is the primary determinant of enterovirus serotype, plays a major role in antigenicity (62), and is the site of receptor binding (15, 17). Therefore, variation in VP1 sequences is likely a contributor to the shifting epidemiology of EV-D68. 2C and 3D^pol^ are also common sites of variation and are key determinants of viral replication. Another notable divergence of contemporary strains from the historical prototypes is contraction of the spacer region in the 5′-UTR, located between the IRES and the open reading frame. This change may be associated with increased activity of the IRES (41, 63). The 5′-UTR is a well-established determinant of virulence in other enteroviruses including poliovirus (64), and therefore, the evolution of the EV-D68 5′-UTR may contribute to increased virulence.

The earliest analysis of EV-D68 molecular evolution came from respiratory disease surveillance in the Netherlands in 1994 to 2010, demonstrating that rising EV-D68 circulation in 2010 coincided with increasing diversity in the VP1 gene sequence (65). Subsequently, samples taken in 2012 to 2014 from patients with AFM in California and Colorado contained six polymorphisms throughout the open reading frame, and interestingly, five of these were synonymous with the sequences of two other neurotropic enteroviruses, poliovirus and EV-D70. All these strains belonged to the newly emergent B1 subclade of EV-D68 (66). A separate study of 2014 strains isolated from patients with respiratory disease in Missouri identified cocirculation of multiple independent lineages largely belonging to the B1 subclade (67). Another study of 2014 US strains found that the B1 subclade was linked to AFM and identified a total of 12 polymorphisms shared with other neurotropic enteroviruses. These were largely concentrated in the 5′-UTR. 2C, VP1, and 3D were the next best represented locations (68). The B3 subclade was first identified in Taiwan and China in 2014 and shares a common ancestor with subclade B1. Strains from subclade B3 had polymorphisms distributed throughout the open reading frame, most commonly in VP1. However, many of the previously identified sites of sequence similarity to neurotropic enteroviruses in subclade B1 were not present (69). Nonetheless, the B3 subclade was dominant in the 2016 and 2018 outbreaks in the United States and Europe, including among patients with AFM (70, 71, 72, 73), and in 2018, all US isolates were from clade B3 (74).

The mechanisms for these changes are predominantly single nucleotide mutations, thought to be random events during the course of viral replication. However, larger rearrangements have occurred, such as the deletion in the 5′-UTR spacer region and at least one incidence of recombination between subclades has been noted (67).

## Tissue tropism and neuropathogenesis

### Tropism

Tropism, the ability of a virus to infect a given cell type or tissue, is an important determinant of disease following viral infection. Enteroviruses have been associated with a wide range of clinical syndromes, including upper and lower respiratory tract infections, GI illness, hand-foot-mouth disease, conjunctivitis, and AFM. These differences are thought to be driven by differing cell surface receptors, whose expression pattern differ among host tissues, and by differing IRES sequences, which are utilized to different degrees in diverse cell types. Therefore, there has been great interest in understanding the molecular basis for the neurotropism of EV-D68.

*In vitro* experiments have demonstrated that EV-D68 can infect multiple cell lines, including HeLa, HTB10, RD, mouse embryonic fibroblasts and the neuron-like cell lines SH-SY5Y and N2a (12, 27). Infection has also been demonstrated in mouse brain organotypic slice cultures, primary mouse astrocytes (27), and multiple nervous system cell types derived from human iPS cell lines, including spinal motor neurons, cortical neurons, and astrocytes (27). In a mouse model of EV-D68 AFM, viral particles were identified within the soma of spinal motor neurons (75). Some controversy exists as to whether neurotropism is unique to contemporary strains of EV-D68. Studies in SH-SY5Y cells showed little to no replication of the historic Fermon-like VR1197 and the 2012 clade A strain USA/N0051U5/2012, in contrast with four 2014 clade B and D strains, which did effectively infect and replicate within these cells (12). By contrast, Fermon, Rhyne, and multiple 2014 EV-D68 isolates infect iPS-derived cortical neurons (27) and spinal motor neurons (26). The discrepancies in neurotropism might be accounted for by different historical EV-D68 strains used in the experiments and the fact that SH-SY5Y cells are not true neurons.

Beyond the nervous system, mouse models of EV-D68 infection have demonstrated broad tropism to multiple tissue types but to varying degrees of intensity. Viral loads were equally high in muscle as in spinal cord and moderately high in lung. Nonzero viral loads were also found in all other tissues studied (75, 76, 77). As for cells derived from the respiratory tract, EV-D68 has been shown to grow within the A549 respiratory carcinoma cell line and primary human upper airway epithelia *in vitro*. It caused toxicity to ciliated cells in the human primary epithelia model, suggesting this cell type may be a particular target of tropism for the primary respiratory infection in human upper airway infection (78). Lower respiratory infection has yet to be investigated in detail.

Overall, it appears that EV-D68 has broad tropism to many neuronal and non-neuronal cell types *in vitro* and *in vivo*, and that neurotropism is not necessarily unique to contemporary strains.

### Neuroinvasion

Neurotropism alone is insufficient for a virus to achieve neurologic infection. It must first spread from the site of primary infection into nervous system tissue, called neuroinvasion. Therefore, *in vitro* evidence alone is insufficient to establish the neuropathogenicity of a given virus. Among picornaviruses, this process is best understood in poliovirus infection. The GI tract is the primary site of infection for poliovirus, where it recognizes its receptor, CD155, on GI epithelial cells. From there, it may establish viremia, entering the systemic circulation *via* the lymphatic system (79). Peripheral tissues may become infected next, most notably muscle. Poliovirus then replicates within and is released from muscle fibers and may cross the neuromuscular junction and enter motor neuron axon terminals through another round of CD155 receptor–mediated endocytosis. It then travels retrograde within endosomes *via* dynein-mediated fast axonal transport to reach the soma of spinal motor neurons (80, 81, 82, 83). Poliovirus particles have been demonstrated in the motor cortex in mouse models of poliomyelitis (84), and corresponding lesions have been described in the motor cortices of patients with poliomyelitis at autopsy (85, 86), indicating that poliovirus can undergo yet another round of trans-synaptic transmission and retrograde transport. A second route for poliovirus neuroinvasion is direct hematologic seeding of the spinal cord across the blood–brain barrier following viremia (87).

A similar paradigm for neuroinvasion has been proposed for EV-D68. In a mouse model of AFM following intramuscular injection of EV-D68, the earliest evidence of viral particles within motor neuron soma was in the neurons innervating the injected muscle. Companion experiments were performed in human iPS-derived motor neurons. Application of EV-D68 only to the axonal compartment of neurons in a microfluidic chamber could lead to detectable virus in the motor neuron soma in an axon length-dependent fashion, and infectious virus could be detected in the culture media of the somatodendritic compartment 24 h later, implying viral replication and release. This process could be prevented by transection of the axon or treatment with the microtubule depolymerizing drug nocodazole (26). These experiments support the hypothesis of retrograde transmission of EV-D68 across the neuromuscular junction, followed by retrograde axonal transport.

**Figure 4:**
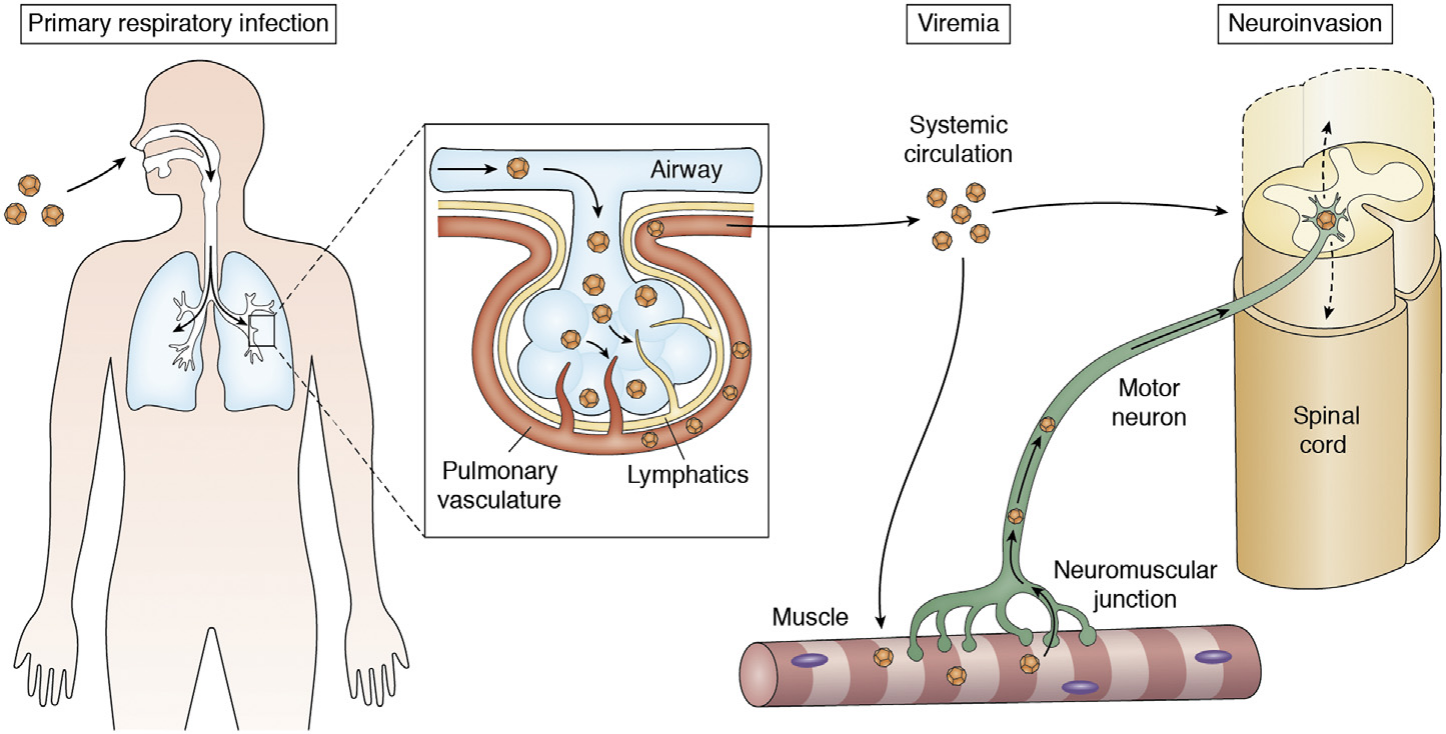
**Hypothesized mechanisms of neuroinvasion.** Enterovirus D68 (EV-D68) is primarily transmitted as a respiratory infection. In a subset of patients, EV-D68 may translocate into the blood stream to establish viremia. The pathway might involve direct entry into the circulation or like poliovirus may occur first *via* the lymphatic system. After establishing systemic circulation, EV-D68 may enter the central nervous system in one of two ways: (1) direct hematogenous seeding or (2) replication in muscle fibers followed by translocation across the neuromuscular junction and retrograde transport within the motor axon. EV-D68 then likely continues to spread within the spinal cord.

Although intramuscular and especially intracerebral routes of experimental infection achieve a high rate of neuropathogenic infection, inoculation of mice by the intranasal route leads to paralysis in only 2.7% of mice (75). This rate is more comparable to observations in humans and implies an indirect route from the nasopharynx to the nervous system. By contrast, no mouse model of AFM has been successfully demonstrated from oral administration of EV-D68. It is hypothesized that EV-D68 progresses from intranasal, to pulmonary, to bloodborne infection before accessing the neuromuscular system, analogous to the transit of poliovirus from oropharynx to systemic infection *via* the GI tract (Fig. 4).

In human AFM patients, the delay between onset of a systemic infectious symptoms and limb weakness (median, 5–7 days) (88, 89) matches the time required for retrograde axonal transport along the length of a typical nerve (10–20 cm/day) (90), and symptoms arise earliest in the most proximal muscles (91). Retrograde transport of EV-D68 from neuromuscular junctions into the anterior horn of the spinal cord may therefore account for these clinical phenomena.

### Neuropathophysiology

MRI of the spinal cord of human AFM patients shows increased signal on T2-weighted sequences, indicative of inflammation or gliosis. This signal is initially present throughout the gray matter and becomes increasingly restricted over time to the anterior horns, the anatomic location of motor neuron cell bodies. This pattern highlights the primary site of central nervous system pathology (92, 93, 94). Electromyography and nerve conduction studies (EMG/NCS) in patients with AFM have also consistently demonstrated findings referrable to motor neuron injury, including diminished or absent compound muscle action potentials, signs of denervation including fibrillations and positive sharp waves, and reduced or absent recruitment of voluntary motor unit potentials. There are no reported EMG/NCS abnormalities of sensory nerve or muscle (95, 96, 97, 98, 99, 100, 101, 102). Electrophysiologic findings generally emerge 1 to 2 weeks after onset of symptoms, concurrent with the transient appearance of gadolinium contrast enhancement in ventral nerve roots on MRI. These clinical observations are consistent with spinal motor neuron injury at the level of the cell body, followed by Wallerian degeneration of motor axons (103, 104).

Cerebrospinal fluid (CSF) samples also show a robust inflammatory response to infection concurrent with the initial diffuse gray matter signal on MRI (91). A mouse model of EV-D68 infection leading to AFM demonstrates viral particles within the soma of spinal motor neurons of the anterior horns and motor neuron death correlating neuroanatomically with limb weakness (75). Collectively, these clinical and animal model data, along with the analogy to poliomyelitis, support a model in which EV-D68 infects motor neurons *in vivo*. It is likely that infection of motor neurons by EV-D68 is directly responsible for motor neuron injury. However, it is unknown whether the inflammatory response to infection is predominantly beneficial in clearing EV-D68 or detrimental in causing further neurologic injury.

The chain of events leading from EV-D68 infection to the dysfunction and/or death of motor neurons resulting in paralysis is largely unstudied. The simplest explanation might be lytic cell death; however, this does not fit the available experimental and clinical observations. *In vitro*, iPS-derived motor neurons were able to sustain multiple rounds of viral infection and replication over the course of 72 h, without apparent cytopathic effect (26). The release of EV-D68 from motor neurons is therefore not obligatorily lytic and may instead progress through the exocytic process described previously. In addition, a well-documented series of EV-A71 AFM patients (105) and scattered anecdotal experience with EV-D68 have demonstrated partial or complete spontaneous reversal of paralysis 1 to 2 months after symptom onset. This time course is faster than can be accounted for by reinnervation of muscle by collateral sprouting from surviving motor axons. Two cases of spontaneous recovery have been characterized by reversal of the patient's EMG/NCS to normal, without electrophysiologic evidence of nerve regeneration or collateral reinnervation (99, 106). Therefore, enterovirus-infected motor neurons likely pass through a reversible stage of dysfunction, after which they may proceed to either cell death or recovery. A similar model has been proposed in poliomyelitis on the basis of pathologic observations in animal models (86).

It is likely that altered host cell biology resulting from the myriad actions of nonstructural proteins of EV-D68 also contribute to cellular death and dysfunction. Notably, many of the cellular processes altered by EV-D68 infection are well characterized to promote motor neuron degeneration in diseases such as amyotrophic lateral sclerosis and spinal muscular atrophy, including RNA metabolism (107, 108), nucleocytoplasmic transport (109), and autophagy (110, 111), and these are discussed in further detail later. Autopsy data from the single patient known to have succumbed in the acute phase of EV-D68 AFM showed spinal cord invasion by T lymphocytes and expression on motor neurons of perforin, a pore-forming cytotoxic protein derived from T lymphocytes. These findings suggest a possible role for cytotoxic T lymphocytes in motor neuron death (112).

To summarize the genetic and experimental data on EV-D68, the key features differentiating it from other enteroviruses and from its own historical prototypes include a unique host receptor, reduced stringency of receptor-binding characteristics, wider range of optimal temperatures, more efficient viral uncoating, more efficient RNA transcription, possible changes in RNA replication, and divergence in sites of antigenicity (Fig. 3). The increasing prevalence of EV-D68 and its association with both severe respiratory disease and neurologic disease may therefore be best explained by generalized changes in virulence and immunogenicity, rather than neurotropism *per se*. It has been proposed that increased virulence leads to more severe infection, greater likelihood of establishing viremia, and therefore a higher rate of neuroinvasion (68).

## Immune responses to infection

### Innate immunity

The first line of defense against enterovirus infection is the innate immune system. Enteroviruses, in general, activate intracellular pattern recognition receptors, including the Toll-like receptors (TLRs), TLR 7/8 (which recognizes genomic ssRNA) and TLR3 (which recognizes the RNA replication intermediate dsRNA) (113, 114). TLR activation initiates a signaling cascade that leads to the production of type I interferons and activation of NF-κB (115). In addition, melanoma differentiation–associated protein (MDA5) and retinoic acid–inducible gene (RIG-I)–like receptors recognize cytoplasmic RNA and induce type I interferons (116, 117, 118). Following TLR activation, nucleotide-binding oligomerization domain–like receptors are activated and incorporated into inflammasomes. The end result of this pathway is activation of caspase-1 resulting in the secretion of proinflammatory cytokines IL-1β and IL-18 (119, 120). MDA5 and TLR3 appear to be the primary drivers of effective innate immunity against enteroviruses in mice (114, 117, 121, 122, 123, 124, 125).

In humans, many enterovirus infections do not proceed to a symptomatic phase and are cleared prior to the production of antibodies. This observation provides additional evidence that innate immunity alone can be sufficient to control viral replication (121, 122, 126). The antiviral role of innate immunity has not been specifically studied for EV-D68. However, in a mouse model of EV-D68 respiratory disease, infection led to robust pulmonary inflammation, airway hyperresponsiveness, and cytokine secretion that exacerbated EV-D68 disease. These data demonstrate that immune responses can also be harmful and provide a potential mechanism for the severe asthma exacerbations seen in children (127).

### Humoral immunity

There is clear evidence of a humoral immune response to EV-D68 in human patients with AFM. An unbiased screen for viral antibodies in CSF collected from 42 AFM patients and 58 controls demonstrated enrichment only for the Picornaviridae family, almost all of which was accounted for by the *Enterovirus* genus. The most commonly identified epitopes were within the VP1 capsid protein and 3D^pol^, respectively. The presence of antibodies against enterovirus VP1 was confirmed by ELISA, demonstrating immunoreactivity in 85% of AFM patients versus 14% of controls (128). A second study was screened for Enterovirus antibodies in CSF from 14 AFM patients and 26 controls also identified a dominant epitope in VP1 (129). These VP1 epitopes overlap with a highly conserved sequence known to induce broad immunoreactivity across the *Enterovirus* genus (130). In addition, antibodies against an EV-D68-specific sequence in a distinct region of VP1 were identified in 43% of CSF and 73% of serum in patients with AFM and none of the controls (129). Of note, each of these studies utilized linear epitopes and therefore cannot identify antigenic sites that require tertiary or quaternary structures or that span multiple capsid proteins. Antigens with these features have been important determinants of immunity to multiple enterovirus species (17, 131, 132, 133, 134). In EV-D68, the crystal structures of four monoclonal antibodies in complex with the virion have been determined, and in all cases, they have spanned multiple capsid proteins or distinct domains of a single protein (135, 136). The prevalence of a humoral response to enteroviruses in patients with AFM is therefore underestimated, and some antigenic epitopes of EV-D68 are probably yet to be identified. Nonetheless, these studies demonstrate intrathecal and systemic antibody production against enteroviruses in patients with AFM, with EV-D68-specific epitopes present in the majority, and identify key viral epitopes responsible for immunogenicity.

Humoral immunity is thought to play a critical role in the prevention and clearance of systemic enterovirus infections. The best example is the robust immunity induced by vaccination against poliovirus and its prevention of poliomyelitis (137, 138). Antibodies appear to play a similarly important role in disseminated coxsackievirus infection in animal models and human patients (139, 140). Further supportive evidence comes from clinical observations in children with X-linked agammaglobulinemia, who lack B cells and are incapable of antibody production. These patients are susceptible to chronic enterovirus infections leading to slowly progressive encephalomyelitis, which may be punctuated by acute relapses that clinically and radiographically resemble AFM. Prophylaxis with intravenous immunoglobulin (IVIG) decreases the incidence of chronic enterovirus encephalomyelitis, and treatment with intravenous and especially intrathecal immunoglobulin may promote resolution of established chronic infection (141). These clinical observations further highlight the importance of humoral immunity in the prevention and/or clearance of central nervous system infection by enteroviruses. Studies of several antibodies generated following EV-D68 infection of mice have demonstrated binding to various sites on the capsid and multiple mechanisms of neutralization, including prevention of receptor binding, premature transition from mature virion to the uncoating intermediate A-particle, or prevention of virus internalization (136, 142).

### Cell-mediated adaptive immunity

T cell-mediated responses to infection are also well documented among enteroviruses. Poliovirus-specific CD4+ T-cell activation has been shown following immunization (143, 144), and these T cells can lyze poliovirus-infected cells (145). Similar observations have been made for coxsackieviruses, but the T cell response has also been implicated in promoting myocarditis as a secondary complication (146). T cell-mediated immunity also appears to be a major factor in determining severity of hand-foot-mouth disease from EV-A71 infection (147). T cell epitopes of EV-D68 have been cataloged from the Immune Epitope Database, indicating that predominantly CD4+ helper T cell epitopes have been studied, and these are largely directed against the structural proteins, with VP1 most strongly represented (148). In a mouse model of EV-D68 respiratory disease, IL-17-dependent cytokine production further supports a role for helper T cell responses (127). The aforementioned autopsy case also highlighted a role for T lymphocytes in the EV-D68 immune response and/or downstream cell death cascades in neurologic disease (112).

## Altered host cell biology

Numerous features of host cell biology are modulated in the course of enterovirus infection. The reason for these alterations appears to be repurposing of host cellular pathways toward the replication and maturation of viral particles or impairment of host immune responses. Except where otherwise specified, the data discussed in this section derive from non-EV-D68 enteroviruses but are hypothesized to apply to EV-D68 as well.

### Innate immunity

Enteroviruses employ multiple strategies for evading the host innate immune response. The earliest line of defense is preventing recognition or subsequent signal transduction by pathogen recognition receptors. The 2A^pro^ of multiple enteroviruses directly cleaves MDA5 (116, 117, 118, 149). Inhibition of MDA5-dependent signaling has also been observed in EV-D68 infection, though appears to be a 3C^pro^-mediated process (150). 3C^pro^ also prevents formation of a functional signaling complex following RIG-I activation (151) and cleaves the TLR3 adapter protein, TRIF (152, 153, 154). The activation of downstream effectors is also attenuated by EV-D68 infection, including 3C^pro^-mediated cleavage of IRF7 (155) and 2C-mediated inhibition of NF-κB activation (156). The end result of inhibiting each of these pathways is diminished production of type I interferon, resulting in reduced host antiviral responses. Host cell RNA translation is also inhibited as discussed in the following section, and this likely reduces interferon production further.

### RNA metabolism

Among the best studied examples of picornavirus subversion of host cell biology is the repurposing of RNA metabolism toward replication of the viral genome. The 3C protease cleaves the TATA box–binding protein (157), cAMP response element–binding protein (158), and Oct-1 (159), thereby inhibiting the action of RNA polymerase II in producing host pre-mRNAs. Maturation and nuclear export of pre-mRNA is also inhibited by the actions of enterovirus proteases. 2A^pro^ cleaves the nucleoporins Nup98, Nup62, and possibly Nup153, whereas 3C^pro^ cleaves Nup214, RanBP2, and Nup153 (160, 161, 162, 163). One result of this attack on the nuclear pore complex is the disruption of the nuclear export of RNA, which is tightly coupled to pre-mRNA splicing and is required prior to translation of RNA in the cytoplasm (164). Pre-mRNA splicing is also directly inhibited by the action of nuclear localized 3D^pol^ on the spliceosome component pre-mRNA substrate factor 8 (Prp8) (165). Finally, 2A^pro^ cleaves eIF4G, an obligatory factor for 5′-cap–mediated translation of mRNA (166, 167). The resulting cleavage product is sufficient to mediate IRES-dependent translation of viral RNA but not cap-dependent translation of host RNA. Host mRNA translation is in fact almost completely shut down during the course of infection (168). Taken together, host RNA metabolism is inhibited in at least three separate stages, allowing the virus to divert critical host resources toward the production of its own RNA and protein.

### Nucleocytoplasmic transport

The aforementioned disruption of the nuclear pore complex does not only affect RNA transport. Multiple nuclear proteins also redistribute to the cytoplasm, many of which are RNA-binding proteins that are repurposed for the translation or replication of viral RNA as described previously. It is not known whether this redistribution of proteins is due to nonspecific leakage of nuclear contents, or a more selective alteration of nucleocytoplasmic transport, though bidirectional redistribution of several nucleocytoplasmic cargoes has been demonstrated following poliovirus infection (169). Given that nuclear RNAs do not leak nonspecifically from the nucleus, and are in fact retained, one might hypothesize that altered protein trafficking results from alteration of specific nucleocytoplasmic transport pathways rather than nonspecific leakage.

Although alterations of RNA metabolism and nucleocytoplasmic transport are conserved across multiple enterovirus species, the extent to which EV-D68 produces these same perturbations remains to be investigated. Furthermore, the impact of these alterations in host cell biology on the pathophysiology of AFM is unknown, though it stands to reason that they could provoke substantial toxicity.

### Autophagy

As described previously, the maturation of EV-D68 particles occurs largely within double membrane–bound organelles derived from autophagosomes. Concurrent with this repurposing of the autophagic compartment for viral reproduction is the prevention of autophagosome-to-lysosome fusion *via* 3C^pro^-mediated cleavage of the autophagic SNARE protein SNAP29, and therefore, native autophagic degradation in the host cell is inhibited (60). In addition, the autophagic adapter protein SQSTM1 is cleaved during the course of EV-D68 infection (60). Related work in coxsackievirus B3 suggests that SQSTM1 cleavage is mediated by 2A^pro^, and that it results in loss of selective autophagy (170). In EV-A71 infection, autophagy was also found to be actively induced by the viral 2BC peptide (171). These studies paint a picture of active mobilization of the autophagy pathway for the purpose of viral reproduction and maturation and away from autophagic degradation. The ultimate impact of these alterations in autophagy by EV-D68 on host cell biology has not yet fully studied; however, it is notable that loss of functional autophagy has well-characterized neurotoxic consequences (172, 173).

### Cell cycle arrest

Many viruses manipulate host cell cycle events to promote viral replication. The EV-D68 3C protein promotes entry into G0/G1 phase, and the 3D protein induces arrest at this phase. There are associated alterations of cyclins and cyclin-dependent kinases. Synchronization of cells in G0/G1 phase promotes the replication of EV-D68 by an undetermined mechanism (174). Interestingly, this is in contrast with EV-A71 and coxsackievirus B3, which arrest the cell cycle at S phase (175), and whose replication is actually inhibited in G0/G1 (174). These results not only indicate the importance of the cell cycle in modulating the susceptibility to enterovirus infection and/or replication and the ability of enteroviruses to manipulate the cell cycle to their benefit but also highlight variability among different enterovirus species.

### Stress granules

Stress granules are punctate structures containing mRNA, ribosomes, translation initiation factors, and RNA-binding proteins that form in the cytoplasm of cells following a variety of stressors, including viral infection (176). Stress granule formation also has antiviral activities, including the sequestration of RNA-binding proteins and translation initiation factors required for the translation and replication of viral RNA.

Stress granules are induced at the onset of enterovirus infection and then rapidly inhibited. This process appears to result primarily from the activity of 2A^pro^ on an as yet undefined target (177). In addition, 3C^pro^ cleaves Ras-GTPase-activating protein SH3-domain-binding protein 1 (G3BP1), an RNA-binding protein that is critical in the formation of stress granules.

Stress granules also form a platform for pattern recognition receptors recognizing viral RNA (178), and the presence of stress granules enhances interferon production following infection by multiple viruses (179, 180). G3BP1 also binds dsRNA and RIG-I and acts as a positive regulator of type I interferon production (181). G3BP1 directly binds to the viral IRES and diminishes its ability to initiate translation (182). Accordingly, G3BP1 expression and/or stress granule formation have been shown to have antiviral effects against multiple enteroviruses (183, 184, 185, 186).

These results have been recently extended to EV-D68. EV-D68 infection leads to transient formation and then dissolution of stress granules and cleavage of G3BP1 (177, 187). Stress granule proteins, G3BP1, TIA1, and HUR, each interact with the 3′-UTR of EV-D68 (+)-ssRNA and diminish viral replication (187).

## Epidemiology

For several decades, EV-D68 infection was considered rare, with only 26 cases identified *via* passive surveillance in the US National Enterovirus Surveillance System from 1970 to 2005 (188), though several small outbreaks associated with respiratory disease were reported worldwide in subsequent years (reviewed in Holm-Hansen *et al.* [189]). The first recognized large outbreak in the United States came in 2014, in which there were a total of 1395 EV-D68 infections confirmed by the US Centers for Disease Control and Prevention (CDC). These cases were recognized through more widespread testing, which was prompted because of increased rates of severe respiratory disease and asthma exacerbations (https://www.cdc.gov/non-polio-enterovirus/about/ev-d68.html). A concurrent outbreak in Canada was detected, numbering 268 confirmed cases (190), followed by reports of increased EV-D68 detection throughout Europe, South America, and Asia.

### Enterovirus D-68 as a causative agent of AFM

The first cases of AFM associated with EV-D68 were reported in a CSF sample from a young adult patient in 2005 (188), and in 2008 in a child who died at home of paralysis and respiratory failure (112). A localized cluster of AFM was next noted in 2012 in California, leading to the first official case definition and initiation of passive surveillance (101). The first major outbreak of AFM occurred in temporal association with the 2014 EV-D68 epidemic, with 120 cases of AFM reported in the United States (https://www.cdc.gov/acute-flaccid-myelitis/cases-in-us.html). This confluence of events raised the possibility that EV-D68 may be the leading causative agent for AFM (191). During that outbreak, the CDC reported that 43% of cases had enterovirus or rhinovirus isolated from respiratory samples, 20% from stool or rectal swab, 2% from CSF, and no cases tested positive from serum or plasma (88).

AFM continued to coincide with outbreaks of EV-D68 in 2016 and 2018, with the United States reporting 160 and 238 confirmed cases, respectively. However, despite this temporal association, few viruses were identified in patient samples, and EV-D68 was only isolated in 20 to 40% of cases (192). This may in part reflect limitations in timing and collection of specimens for testing, as samples collected closer to the onset of respiratory or febrile illness are more likely to test positive for EV-D68 (193). Despite the challenges in identifying the causative agent in a majority of patients with AFM, additional lines of evidence point toward EV-D68. Two independent studies of sera and CSF from patients with AFM demonstrated strong enrichment for enterovirus-associated antibodies, including antibodies against an EV-D68-specific epitope in up to 73% of patients (128, 129). In addition, multiple mouse models of EV-D68 have been developed that replicate key features of AFM (27, 75, 77, 194, 195), and Koch's postulates for causation were fulfilled in one mouse model (75). Two recent analyses have concluded that the Bradford Hill criteria support a causative relationship between EV-D68 and AFM in humans (196, 197). Other enteroviruses and rhinoviruses are also associated with AFM at lower rates, most notably EV-A71 and coxsackievirus A16 (193).

### Children are at greatest risk of AFM

The median age at presentation reported in the United States and Europe from 2014 to 2018 ranged from 4.0 to 7.8 years. Though the age range of cases extends into young adulthood, the vast majority of cases have been in children younger than 10 years (192, 198). EV-D68 infections are thought to be widespread, as suggested by a high rate of mild infections found on prospective surveillance (74). Based on these data, the prevailing expert opinion is that the attack rate for AFM following EV-D68 infection is very low, perhaps less than 1%, though data to quantitatively establish the attack rate are lacking. For comparison, the attack rate of poliomyelitis following infection with poliovirus is estimated to be 0.1% (199).

### Seasonality

Enteroviruses have seasonal incidence patterns in temperate and tropical climates (200). Infections caused by enteroviruses usually peak in the late summer and early fall in the United States (188, 201, 202). Passive surveillance systems like the US National Enterovirus Surveillance System and the National Respiratory and Enteric Virus Surveillance System indicate that EV-D68 peaks occurred in 2014 and 2016 and accounted for 56% of the reports of *Enterovirus* and *Parechovirus* (total n = 2967). Mathematical modeling of nonpolio enterovirus incidence implicates climate, specifically the dew point temperature and latitude, as the most important factors driving seasonality of enteroviruses. Birth rates are an additional factor driving the amplitude of each peak (203). However, without active surveillance, it is difficult to determine circulation of enteroviruses across age populations.

In addition to seasonal variation, EV-D68 established a biennial pattern of outbreaks from 2012 to 2018. This appears to be explained by fluctuating levels of susceptibility in the population, with each wave of EV-D68 infection generating temporary herd immunity that wanes as new nonimmune children grow into the susceptible age range. This hypothesis is backed by mathematical modeling data (204) and parallels patterns that have been observed in other enterovirus species (205). Biennial circulation was disrupted in 2020, likely a secondary effect of public health measures instated to control the coronavirus disease 2019 (COVID-19) pandemic.

### Host genetic risk factors for AFM

The low attack rate of AFM following EV-D68 infection implies that viral infection alone is not sufficient to explain neuroinvasive infection. Instead, host factors and/or environment are likely permissive. Within regions, AFM is rare and not clustered around a city, town, or school. AFM typically affects only one family member in any household despite multiple family members reporting symptoms or testing positive for EV-D68 infections. These patterns suggest that there is not an environmental factor inducing the severe neurologic outcome, or there would be more readily apparent clustering of cases. Instead, AFM seems to fit genetic models of rare autosomal recessive inheritance or *de novo* mutations. Direct evidence of genetic susceptibility to neurovirulent infection exists in poliomyelitis, wherein there is a higher rate of disease among siblings of affected children (including across separate outbreaks), and the rate of concordance is higher among monozygotic than dizygotic twins (206, 207). Similar studies have not been possible in AFM because of the much lower number of cases. The specific genetic determinants of susceptibility to polio have not been determined, though it is possible that they are shared with AFM. A better understanding of the relationship between host genetics and AFM could help elucidate pathogenic mechanisms and identify putative therapeutic targets.

## Potential therapeutic approaches

Currently, there are no proven therapies for EV-D68 infections. For respiratory disease, supportive care is provided, in addition to management of asthma exacerbation when present. The mainstay of treatment in AFM is also supportive care, along with early initiation of aggressive rehabilitative therapies (208). In addition, IVIG has become the standard of care despite a lack of controlled clinical trials (209). This practice is based on the observation that commercial preparations of IVIG contain high titers of neutralizing antibodies against EV-D68 (210) and the rationale that IVIG administration may confer passive immunity to limit the progression or enhance the clearance of viral infection. IVIG also has anti-inflammatory effects that are of unknown benefit for management of AFM. The use of IVIG in a mouse model of EV-D68 AFM was protective against paralysis but only if given very early in the course of infection (211). A monoclonal antibody against EV-D68 showed a similar pattern of protection in mice (135). Immune suppressive therapies have also been used by some centers to target the inflammatory response to infection. However, in the EV-D68 mouse model of AFM, steroid treatment markedly exacerbated the severity of disease, especially when given early postinfection (211). In addition to these acute therapies, there is ongoing interest in the development of a vaccine for EV-D68, and monoclonal antibodies have been proposed as either acute or preventive treatment. Several efforts at these strategies are underway (77, 135, 136, 212, 213).

Antiviral pharmacologic therapies have also been proposed, targeting numerous facets of the viral life cycle. Among the most commonly investigated strategies are capsid-binding drugs, which prevent receptor binding and/or internalization of viral particles (19, 214, 215, 216, 217). Other strategies that have shown at least some efficacy against viral replication *in vitro* include protease inhibitors targeting 2A^pro^ or 3C^pro^ (215, 216, 218, 219), 2C inhibitors (194, 215, 220), drugs targeting 3A or its host-derived interacting partners in the replication organelle (214, 215, 216, 221), and inhibition of the RNA-dependent RNA polymerase 3D^pol^ (214, 216, 222, 223). Other approaches have targeted IRES-dependent translation, rather than viral proteins *per se* (224). Please see the recent comprehensive review of EV-D68 antivirals for discussion of specific pharmacotherapies (225).

Several of the most promising antiviral agents from *in vitro* studies have been evaluated in animal models with mostly discouraging results. One study demonstrated efficacy of the 2C inhibitor guanidine for respiratory and neurologic symptoms of EV-D68 infection in mice but at a dose markedly in excess of the established dosing range in humans. There was no efficacy of the capsid-binding agents, pleconaril or pirodavir, 3C^pro^ inhibitor rupintrivir, 2C inhibitor fluoxetine, or the 3A inhibitor enviroxime, though in some cases optimal dosing was not achieved and the mouse model used was incompletely characterized (194). A separate mouse model also demonstrated no efficacy of fluoxetine (211). Another 3C^pro^ inhibitor labeled compound 10 had a favorable pharmacologic profile in rats and inhibited viral replication *in vivo*, but the effect on disease-relevant outcomes was not assayed (219). The sole drug to be evaluated in humans is fluoxetine, which showed no benefit in a retrospective cohort study of AFM (226).

Collectively, these studies demonstrate that therapies that enhance the immune response to EV-D68 or that target the viral replication cycle may be of benefit. Most drug candidates evaluated to date have been repurposed from development efforts for other RNA viruses rather than being tailor made for EV-D68. Even those candidates with *in vitro* efficacy have failed to demonstrate similar efficacy in animal models or human patients or have been limited because of concerns over safety or pharmacokinetics. Animal models of EV-D68 utilize several strategies to ensure reproducible rates of paralytic illness, including nonphysiologic routes of viral infection (194, 211), and genetic knockout of innate immune responses (194). These strategies not only improve the efficiency of the models but also generate caveats that will need to be carefully considered in matching therapeutic candidates to animal models for preclinical testing.

Further complicating the approach to treatment, patients with AFM typically present with paralysis a week or more after the onset of EV-D68 infection (91). At this point, there has already been systemic spread of virus and neuroinvasion. Therapies that target viral replication or the immune response may have little benefit at this late stage of infection. Therefore, strategies targeting the host side of the equation, such as abnormal motor neuron biology and subsequent cell death pathways, may also be of value to prevent or reverse the progression of paralysis in patients with AFM.

## Conclusions

EV-D68 is recently responsible for increasing number of severe respiratory infections and permanent neurologic disability in thousands of children worldwide. Strategies to prevent and treat EV-D68 infections are an urgent public health need. Initial investigations of EV-D68 virology suggests that these trends are due in part to shifting antigenicity and increased virulence, allowing for broader spread of the virus, more severe respiratory complications, and a higher attack rate for neurologic disease in comparison to many other enteroviruses or to its own historic prototype strains. However, much remains to be discovered, including the identity of the primary host receptor for EV-D68 infection and nearly all aspects of motor neuron biology following EV-D68 infection. A detailed understanding of EV-D68 biology will be imperative to promote the efficient development of vaccines, antivirals, and neuroprotective strategies.

Epidemiologic data have demonstrated a consistent biennial pattern of EV-D68 outbreaks since 2012 predominantly not only in the United States but also extending to many parts of the world. These patterns suggest a high likelihood of continuing outbreaks. With regard to its transmission, propensity for paralytic disease, and epidemiologic patterns, EV-D68 bears a strong resemblance to poliovirus, which progressed to epidemic levels within a generation of emergence (227).

A recurrent outbreak of EV-D68 and AFM was predicted for the summer and fall of 2020. However, widespread social distancing and facial mask usage as a result of the COVID-19 pandemic has altered these patterns substantially. Like severe acute respiratory syndrome coronavirus 2, the causative agent of COVID-19, EV-D68 is a respiratory pathogen spread primarily by respiratory droplets, and its transmission was likely reduced by these same measures. This phenomenon has already been noted for several other seasonal respiratory viruses (228, 229), and 2020 surveillance data from the US CDC show only 29 confirmed cases of AFM as of January 4, 2021, a rate that is comparable to off-peak years when EV-D68 outbreaks are not detected (https://www.cdc.gov/acute-flaccid-myelitis/cases-in-us.html). These changes may disrupt the biennial pattern of circulation. On the other hand, the increased numbers of nonimmune children resulting from the absence of a 2020 outbreak may predispose the population to an even larger outbreak in 2021 and a resumption of recurrent outbreaks. In either case, continued vigilance and ongoing research will be needed to understand and counter this emerging threat.

## Conflict of interest

The authors declare that they have no conflicts of interest with the contents of this article.
